# A Neutralizing RNA Aptamer against EGFR Causes Selective Apoptotic Cell Death

**DOI:** 10.1371/journal.pone.0024071

**Published:** 2011-09-06

**Authors:** Carla Lucia Esposito, Diana Passaro, Immacolata Longobardo, Gerolama Condorelli, Pina Marotta, Andrea Affuso, Vittorio de Franciscis, Laura Cerchia

**Affiliations:** 1 Istituto per l'Endocrinologia e l'Oncologia Sperimentale del CNR “G. Salvatore”, Naples, Italy; 2 Dipartimento di Biologia e Patologia Cellulare e Molecolare, University of Naples “Federico II”, Naples, Italy; 3 Facoltà di Scienze Biotecnologiche, University of Naples “Federico II”, Naples, Italy; 4 Animal Model Facility, Biogem s.c.a.r.l., Ariano Irpino, Avellino, Italy; 5 Stazione Zoologica Anton Dohrn, Naples, Italy; National Taiwan University Hospital, Taiwan

## Abstract

Nucleic acid aptamers have been developed as high-affinity ligands that may act as antagonists of disease-associated proteins. Aptamers are non immunogenic and characterised by high specificity and low toxicity thus representing a valid alternative to antibodies or soluble ligand receptor traps/decoys to target specific cancer cell surface proteins in clinical diagnosis and therapy. The epidermal growth factor receptor (EGFR) has been implicated in the development of a wide range of human cancers including breast, glioma and lung. The observation that its inhibition can interfere with the growth of such tumors has led to the design of new drugs including monoclonal antibodies and tyrosine kinase inhibitors currently used in clinic. However, some of these molecules can result in toxicity and acquired resistance, hence the need to develop novel kinds of EGFR-targeting drugs with high specificity and low toxicity. Here we generated, by a cell-Systematic Evolution of Ligands by EXponential enrichment (SELEX) approach, a nuclease resistant RNA-aptamer that specifically binds to EGFR with a binding constant of 10 nM. When applied to EGFR-expressing cancer cells the aptamer inhibits EGFR-mediated signal pathways causing selective cell death. Furthermore, at low doses it induces apoptosis even of cells that are resistant to the most frequently used EGFR-inhibitors, such as gefitinib and cetuximab, and inhibits tumor growth in a mouse xenograft model of human non-small-cell lung cancer (NSCLC). Interestingly, combined treatment with cetuximab and the aptamer shows clear synergy in inducing apoptosis *in vitro* and *in vivo*. In conclusion, we demonstrate that this neutralizing RNA-aptamer is a promising bio-molecule that can be developed as a more effective alternative to the repertoire of already existing EGFR-inhibitors.

## Introduction

The EGFR/ErbB family of receptor tyrosine kinases (RTK) comprises four members: EGFR (also known as HER1 or ErbB1), ErbB2 (Neu, HER2), ErbB3 (HER3) and ErbB4 (HER4), containing an extracellular ligand binding region, a single membrane-spanning region and an intracellular tyrosine-kinase-containing domain. Unlike the rest of the ErbB family, ErbB3 lacks tyrosine kinase activity and ErbB2 has no known ligand. Epidermal growth factor (EGF) and transforming growth factor-α bind directly only to EGFR, whereas neuregulins (also known as heregulins) are specific for ErbB3 and ErbB4 [1], [2]. Ligand-induced activation of EGFR by dimerization mediates multiple intracellular pathways that inhibit apoptosis and promote survival and proliferation [3], [4].

Deregulation of EGFR by over-expression or constitutive activation promotes tumor processes including angiogenesis and metastasis and is associated with poor prognosis in many human malignancies including glioma, lung and breast cancer [1], [2].

The prevalence of this receptor in well-established cancers has elicited several studies and discoveries leading to the generation of multiple Food and Drug Administration (FDA) approved agents including the monoclonal antibodies (as cetuximab and panitumumab for the treatment of colorectal cancer, NSCLC, and squamous cell carcinoma of the head and neck) that bind to the extracellular domain of EGFR, and small-molecule inhibitors (as gefitinib, erlotinib and lapatinib for the treatment of NSCLC, breast and pancreas cancers) that compete with ATP for binding to the tyrosine kinase domain of the receptor [2], [5]–[8].

The treatment of tumor cells with these agents affects many of the intracellular pathways that are essential for cancer development and progression. However, patients receiving these treatments often show primary or acquired resistance to the inhibitors [9]–[11]. Thus, new strategies to overcome tyrosine kinase inhibitors (TKI) resistance are under active exploration and there is the urgent need to design new EGFR-targeting drugs for a more specific and selective tumor therapy.

An emerging new class of targeted therapeutic molecules against RTKs is composed of nucleic acid-based aptamers. They are short structured single-stranded RNA or DNA ligands that bind with high selectivity and sensitivity, due to their specific three-dimensional shapes, to their target molecules. Aptamers possess many advantages over proteins as therapeutic reagents, including low cost, convenient synthesis and modification with high batch fidelity, no immunogenicity, rapid tissue penetration and long-term stability [12]–[14]. Further, in the last years, aptamers targeting cell surface proteins are being explored as promising delivery vehicles to target a distinct disease or tissue in a cell-type specific manner [13], [15], [16]. The list of inhibitory aptamers for therapeutic use is growing rapidly and one aptamer (Macugen) against the vascular endothelial growth factor was approved by FDA for the treatment of age-related macular degeneration [17].

To date, unmodified RNA-aptamers have been selected against the purified extracellular domain of EGFR and then used for gold-nanoparticles delivery to cancer cells [18] or, in a surface-immobilized form, to capture EGFR-expressing glioma cells [19]. These studies offer solid proof-of-concept for delivery strategies and for cancer cells isolation approaches, even though no direct inhibition of EGFR has been shown. More recently, a 2′-fluoro-modified RNA aptamer against recombinant human EGFR protein has been described [20]. The aptamer blocks EGF-dependent receptor autophosphorylation *in vitro* but has not been evaluated in animals.

Herein, we have generated a nuclease-resistant RNA-aptamer (named CL4) able to bind at high affinity to EGFR on the surface of different cancer cells and to block EGFR downstream signaling *via* inhibition of either EGFR homodimers and heterodimers with cognate ErbB2 or ErbB3, thus irrespective of the ligand that causes receptors dimerization. It induces selective cell death *in vitro* and *in vivo*, thus revealing an EGFR-drug candidate with promising translational potential.

## Results

### CL4 aptamer specifically interacts with EGFR

**Figure 1:**
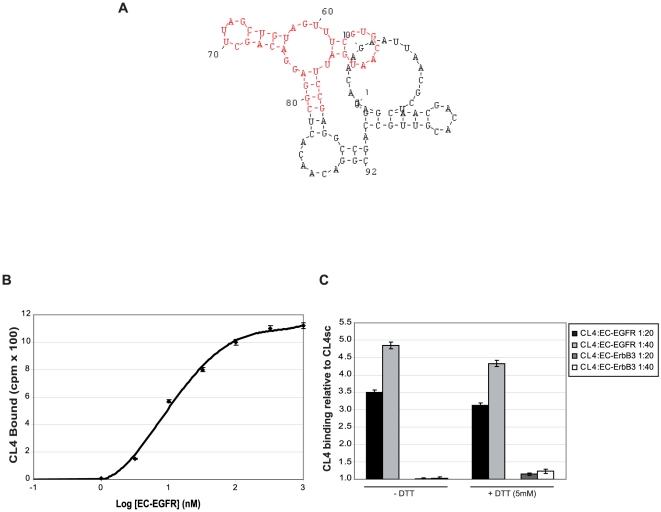
The anti-EGFR CL4 aptamer. (A) Secondary structure of CL4 Full Length predicted by using MFOLD software version 3.1. The structure of CL4 (nucleotides from 42 to 81) is shown in red. (B) Binding isotherm for CL4∶EC-EGFR complexes. K_d_ value was derived by fitting bound CL4 *versus* the protein concentration to the equation Y = BmaxX/(K_d_+X), where Bmax is the extrapolated maximal amount of RNA∶protein complex bound. The specific binding was determined by subtracting the background values obtained with CL4sc from the values obtained with CL4. (C) EC-EGFR or EC-ErbB3 (20 and 40 nM, with and without DTT treatment) were incubated with 1 nM CL4 and radiolabeled protein-bound RNA was collected by nitrocellulose filters and quantified.

By using differential whole-cell SELEX on human NSCLC we identified a set of five families of sequence related 2′-fluoro pyrimidines (2′-F Py) RNA-aptamers that distinguish A549 cells (resistant to cell death induced by TRAIL, cisplatin and paclytaxel) from the more sensitive H460 cells. CL4 full-length (FL), the best candidate aptamer from this selection, efficiently binds to A549 cells with an apparent dissociation constant (K_d_) value of 46 nM (not shown). Based on the predicted secondary structure of the 92mer original molecule, we designed a shorter aptamer of 39mer (herein indicated as CL4) containing the functional site of CL4 FL (Fig. 1A) that preserves high binding affinity to A549 cells with a K_d_ of 38 nM and discriminates them from H460 cells (not shown). Further, using a phospho-RTK array analysis to identify functional targets of CL4 provided us convincing evidence that the target of the aptamer could likely be EGFR and/or ErbB3 (Fig. S1).

To definitely identify the cellular target of CL4 we first performed a filter binding analysis with the soluble extracellular domain of human EGFR and ErbB3 (indicated as EC-EGFR and EC-ErbB3, respectively) as targets, that confirmed a strong affinity of CL4 for EC-EGFR (K_d_ value of 10 nM, Fig. 1B) while no appreciable CL4 binding was observed to EC-ErbB3 (Fig. 1C). Further, CL4 shows comparable binding for both the disulfide-linked EGFR dimer and for the reduced monomer (Fig. 1C).

**Figure 2:**
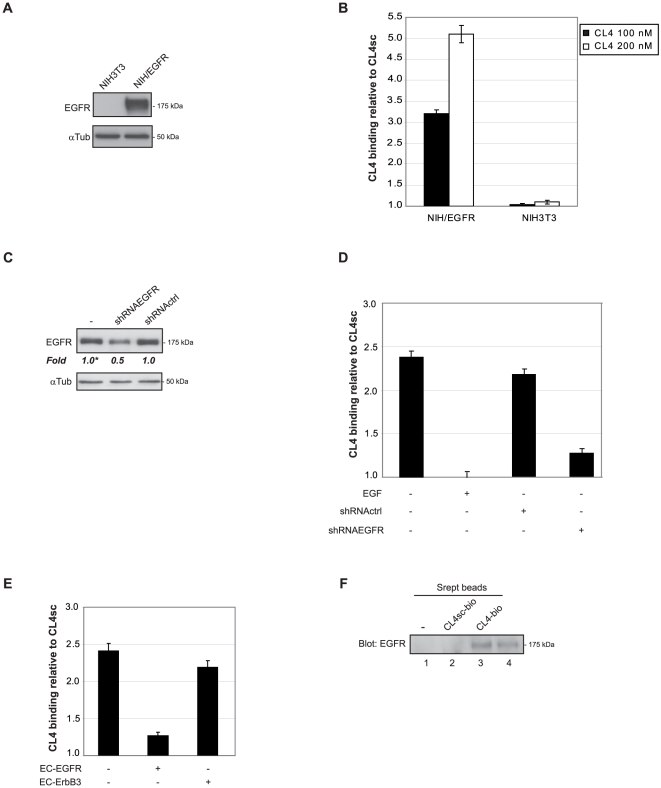
CL4 specifically interacts with EGFR. (A) Lysates from NIH3T3 or NIH/EGFR cells were immunoblotted with anti-EGFR antibodies. αtubulin was used as an internal control. (B) Binding of radiolabeled CL4 on NIH3T3 or NIH/EGFR. (C) Lysates from A549 cells following 72 h-transfection with a specific EGFR shRNA (shRNAEGFR) or a non-related shRNA (shRNActrl) were immunoblotted with anti-EGFR antibodies. αtubulin was used as an internal control. Values below the blot indicate signal levels relative to control non transfected, arbitrarily set to 1 (labeled with asterisk). Intensity of bands has been calculated using the NIH Image Program on at least two different expositions to assure the linearity of each acquisition. (D) Binding of 100 nM radiolabeled CL4 on A549 cells in the absence or in the presence of 1 µM EGF or on A549 cells following 72 h-transfection with shRNAEGFR or shRNActrl. (E) Binding of 100 nM radiolabeled CL4, prior incubated with 300 nM EC-EGFR or EC-ErbB3 for 15 min at 37°C, on A549 cells. In (B–E), the results are expressed relative to the background binding detected with the CL4sc used as a negative control. Error bars depict means ± s.d. (n = 3). (F) Lysates from A549 cells untreated (lane 1) or treated with biotinylated CL4sc (CL4sc-bio, lane 2) or CL4 (CL4-bio, lane 3) were purified on streptavidin beads and immunoblotted with anti-EGFR antibodies. Lane 4, 10 µg-cell lysates. In (A, C, F), molecular weights of indicated proteins are reported.

Accordingly, binding analyses on NIH3T3 cells stably transfected with human EGFR (NIH/EGFR) that express a very high level of EGFR (Fig. 2A) showed that CL4 bound to NIH/EGFR but not to parental cells (Fig. 2B) with an apparent K_d_ value of 60 nM (not shown). Conversely, binding to A549 cells was decreased by interfering with EGFR expression and by high concentration of EGF (Fig. 2C,D). Consistently with its ability to specifically bind to membrane-bound as well as to the soluble ectodomain of EGFR, we found that CL4 binding to A549 cells was competed by EC-EGFR but not by EC-ErbB3 (Fig. 2E).

The specific interaction of CL4 with EGFR on cell surface was further analyzed by affinity purification on streptavidin coated beads of extracts from A549 cells treated with biotin-labeled CL4 followed by immunoblotting with anti-EGFR antibodies. As shown, CL4 specifically interacts with EGFR on cell surface whereas no binding was obtained with CL4sc, the scrambled 2′F-Py RNA used as a negative control (Fig. 2F).

Further, we determined binding of CL4 on stable tumor derived cell lines that differently express EGFR family members (EGFR, ErbB2, ErbB3, or ErbB4). As expected, CL4 binding was detected solely on cells that express high level of EGFR, either in the presence or in the absence of other members of that family (Fig. S2).

Taken together, these results indicate that the CL4 aptamer recognizes specifically and at high affinity the EGFR in its physiological context on the cell surface as well as the purified extracellular domain of the receptor both as monomer or dimer.

### CL4 inhibits EGFR-mediated signaling pathways

Ligand binding to EGFR induces the formation of receptor homo- and heterodimers with three other ErbB family members resulting in kinase activation, tyrosine-phosphorylation and intracellular signaling. Among the EGF family of growth factors, binding of EGF to EGFR preferentially induces the formation of EGFR-EGFR and EGFR-ErbB2 complexes and, at a less extent, of EGFR-ErbB3 [1], [21].

**Figure 3:**
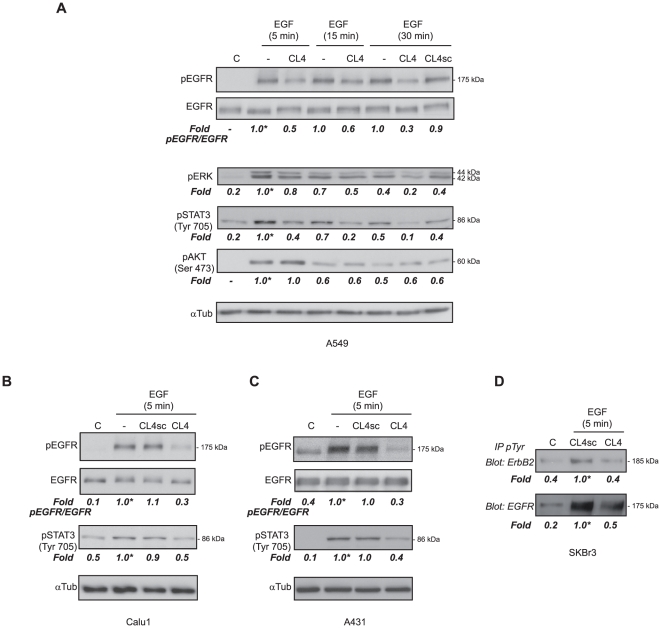
CL4 inhibits EGF-dependent EGFR activation. (A) Serum starved A549 cells (150,000 cells per 3.5-cm plate) were either left untreated or treated for 3 h with 200 nM CL4 or CL4sc and then stimulated for the indicated times with EGF (50 ng/ml) alone or in the presence of each aptamer. Cell lysates were immunoblotted with anti-(phospho)-EGFR (pEGFR), anti-EGFR, anti-(phospho)-ERK1/2 (pERK), anti-(phospho)-STAT3 (pSTAT3), anti-(phospho)-AKT (pAKT) antibodies, as indicated. (B,C) Calu 1 and A431 cells were treated as in (A) and cell lysates were immunoblotted with anti-pEGFR, anti-EGFR and anti-pSTAT3 antibodies, as indicated. In (A–C), αtubulin was used as an internal control. Values below the blots indicate signal levels relative to 5 min-EGF stimulated control, arbitrarily set to 1 (labeled with asterisk). (D Lysates from SKBr3 cells treated as in (A), were immunoprecipitated with anti-(phospho)-tyrosine (pTyr) antibodies and immunoblotted with anti-ErbB2 and anti-EGFR antibodies, as indicated. Values below the blots indicate signal levels relative to 5 min-EGF stimulated CL4sc control, arbitrarily set to 1 (labeled with asterisk). In (A–D), molecular weights of indicated proteins are reported, “C” indicates mock-treated cells. Quantitation was done as in Figure 2. Blots shown are representative of at least four independent experiments.

Thus, we determined in A549 cells whether CL4 could affect EGFR activation following EGF stimulation. CL4 treatment drastically reduced the tyrosine-phosphorylation of EGFR in a time-dependent manner reaching 70% inhibition at 30 min of EGF treatment, whereas no effect was observed in the presence of CL4sc scrambled sequence (Fig. 3A).

Among the main intracellular effectors of EGFR that mediate induction of cell proliferation and resistance to apoptosis are extracellular-signal regulated kinase 1/2 (ERK1/2), AKT and signal transducers and activator of transcription (STAT) proteins [1], [4]. As shown in figure 3A, the phosphorylation of ERK1/2 is strongly induced by EGF with a peak at 5 min and rapidly declines. CL4 treatment reduced the extent of phospho-ERK1/2 at all time points with a maximum of inhibition at 30 min (50% inhibition as compared to CL4sc).

EGF binding to EGFR results as well in the activation of the anti-apoptotic STAT3 protein [1], [22], [23]. We thus looked whether, as a consequence of EGFR inhibition, CL4 could interfere with STAT3 activation. Treatment with CL4 for 15 min was sufficient to inhibit tyrosine-phosphorylation close to the basal level thus showing an inhibition kinetic more rapid for STAT3 than for ERK1/2 (Fig. 3A). Further, in agreement with its ability to bind Calu1 and A431 cells, CL4 strongly inhibited EGF-induced activation of EGFR and STAT3 in these cells (Fig. 3B,C).

On the other hand, even though the phosphorylation of AKT is strongly induced by 5 min-EGF stimulation and rapidly declines, 200 nM-CL4 treatment had no appreciable effect on that kinase and the levels of phospho-AKT Ser473 remained comparable to the control up to 30 min of treatment (Fig. 3A). The same result was obtained looking at phospho-AKT Thr308 (not shown). Increasing the CL4 concentrations up to 500 nM was as well ineffective in inhibiting phospho-AKT levels (unpublished data). A plausible explanation for this unexpected result could be that CL4 preferentially inhibits the EGFR-EGFR homodimers that primarily occur following EGF stimulation. Indeed, the EGFR-dependent activation of phosphoinositide 3-kinase (PI3K)/AKT occurs mainly through dimerization of EGFR with ErbB3 since PI3K docking sites are absent on EGFR and ErbB2, whereas, are highly prevalent on ErbB3 [1], [4].

We thus wondered whether, as a result of its binding to EGFR, CL4 could interfere with ligand-dependent EGFR/ErbB2 heterodimerization in SKBr3 cells that, differently from the analysed NSCLC and A431 cells, express very high levels of ErbB2 (Fig. S2A,C). As shown in Fig. 3D, CL4-treatment resulted in a strong reduction of ligand-induced phosphorylation of ErbB2 that reached the basal, un-stimulated, levels.

**Figure 4:**
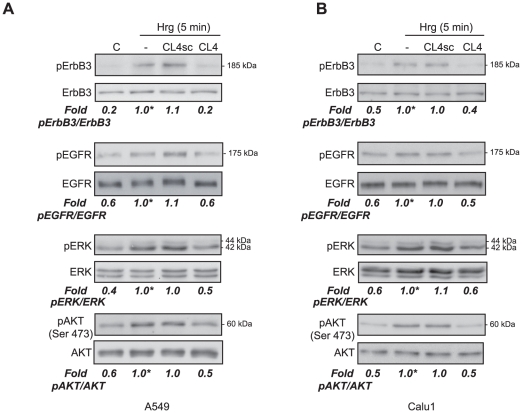
CL4 inhibits Hrg-dependent EGFR activation. (A,B) Serum starved A549 and Calu1 cells (150,000 cells per 3.5-cm plate) were either left untreated or treated for 3 h with 200 nM CL4 or CL4sc and then stimulated for 5 min with Hrg (100 ng/ml) alone or in the presence of each aptamer. Cell lysates were immunoblotted with anti-(phospho)-ErbB3 (pErbB3), anti-ErbB3, anti-pEGFR, anti-EGFR, anti-pERK, anti-ERK, anti-pAKT and anti-AKT antibodies, as indicated. Values below the blots indicate signal levels relative to Hrg stimulated control, arbitrarily set to 1 (labeled with asterisk). Molecular weights of indicated proteins are reported, “C” indicates mock-treated cells. Quantitation was done as in Figure 2. Blots shown are representative of at least four independent experiments.

EGFR activation may also occur upon heregulin (Hrg) binding to ErbB3 and consequent heterodimerization of ErbB3 with EGFR [1], [21]. Thus, in order to investigate whether CL4 may interfere as well with EGFR/ErbB3 heterodimerization, we looked at the effect of the aptamer on the Hrg-mediated signal pathways. To this aim, A549 and Calu1 cells were stimulated with Hrg in the presence of CL4 or CL4sc and the tyrosine-phosphorylation of ErbB3 and EGFR was determined by immunoblotting (Fig. 4A,B). The low intensity of phospho-EGFR after Hrg stimulation likely reflects the low levels of ErbB3 in these cells (Fig. S2A) and suggests that Hrg induces formation of EGFR-ErbB3 heterodimers poorly, in agreement with previously published findings [24].

In the presence of CL4, phosphorylation levels of both ErbB3 and EGFR are reduced to the un-stimulated levels (Fig. 4A,B). Further, CL4 reduced of about 50%, as compared to CL4sc, the Hrg-dependent activation of ERK1/2 and AKT in both the analyzed cell lines (Fig. 4A,B), thus indicating that the action of CL4 on AKT may depend on the specific dimer formed.

The above results indicate that the binding of CL4 to cell-surface exposed EGFR results in blocking of the receptor activation, both if induced by homodimerization and heterodimerization with either ErbB2 or ErbB3, thus in turn hampering the EGFR-dependent downstream signaling pathways. In all cases, inhibition is mediated by specific recognition of EGFR since CL4 has no effect on MCF7 and H460 cells (not shown) that express low or undetectable levels of EGFR (Fig. S2A,C).

### CL4 strongly induces apoptosis in EGFR-positive cells

The identification of an aptamer that specifically binds and inhibits EGFR, hampering the anti-apoptotic STAT3 pathway, raises the obvious question of whether this aptamer may interfere with survival of target cells. The 24 h-treatment of A549, Calu1 and A431 cells with CL4 strongly inhibited cell viability that was reduced of about 60% by comparison with cells untreated or treated with CL4sc. Accordingly with the inability of CL4 to efficiently bind to H460 cells, it did not affect cell viability (Fig. S3A).

Moreover, in our experimental condition no additive effect on A549 cell viability was observed by combining CL4 treatment with TRAIL, cisplatin or paclytaxel (Fig. S3B).

**Figure 5:**
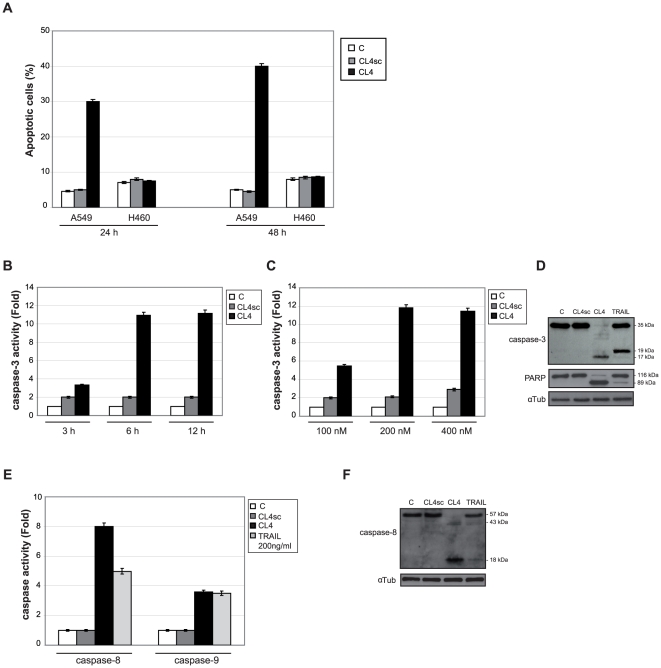
CL4 induces apoptosis. (A) A549 and H460 cells (5,000 cells/well in 96-well plates) were either left untreated or treated for 24 h and 48 h with 3 µM CL4 or CL4sc, renewing treatment each 24 h and the percentage of apoptotic cells (sub-G1 peak) was determined by FACS following PI incorporation. (B,C) A549 were left untreated or treated with 200 nM-final concentration of CL4 or CL4sc for the indicated incubation times (B) or for 6 h with increasing amounts of each aptamer (C) and cell lysates were analyzed by caspase-3 activation fluorimetric assay. (D) Lysates from A549 cells left untreated or treated for 6 h with 200 nM of indicated aptamers or 200 ng/ml TRAIL were immunoblotted with anti-caspase-3, anti-PARP and anti-αtubulin antibodies, as indicated. (E,F) Cell lysates as in (D) were analyzed by caspase-8 and caspase-9 activation fluorimetric assays (E) or by immunoblotting with anti-caspase-8 and anti-αtubulin antibodies, as indicated (F). In (D,F), “C” indicates mock-treated cells. Blots shown are representative of at least three independent experiments. Molecular weights of full-length and cleaved caspase-3, 8 and PARP are reported. In (A–C,E), error bars depict means ± s.d. (n = 3).

In order to dissect the molecular mechanism of CL4 induced cell viability reduction, we analyzed apoptosis in A549 cells following aptamer treatment. Remarkably, we found that the percentage of apoptotic cells was about 30% after 24 h-CL4 treatment reaching 40% after 48 h. No effect was observed in the presence of the negative control (Fig. 5A). Next, we examined whether CL4 treatment activated the executioner caspase-3 and the initiator caspases-8 and 9. As shown, treatment of A549 cells with CL4 resulted in a strong caspase-3 (Fig. 5B–D), caspase-8 (Fig. 5E,F) and caspase-9 (Fig. 5E) cleavage. Cleavage of Poly (ADP-ribose) polymerase (PARP) also correlated with caspase-3 activation (Fig. 5D). High concentration of TRAIL (200 ng/ml) was used as a positive control of caspases activation (Fig. 5D–F).

These data indicate that CL4 interferes with cell proliferation by activating apoptosis, likely by down regulating STAT3 function or that of other members of the same protein family.

### CL4 inhibits tumor growth

**Figure 6:**
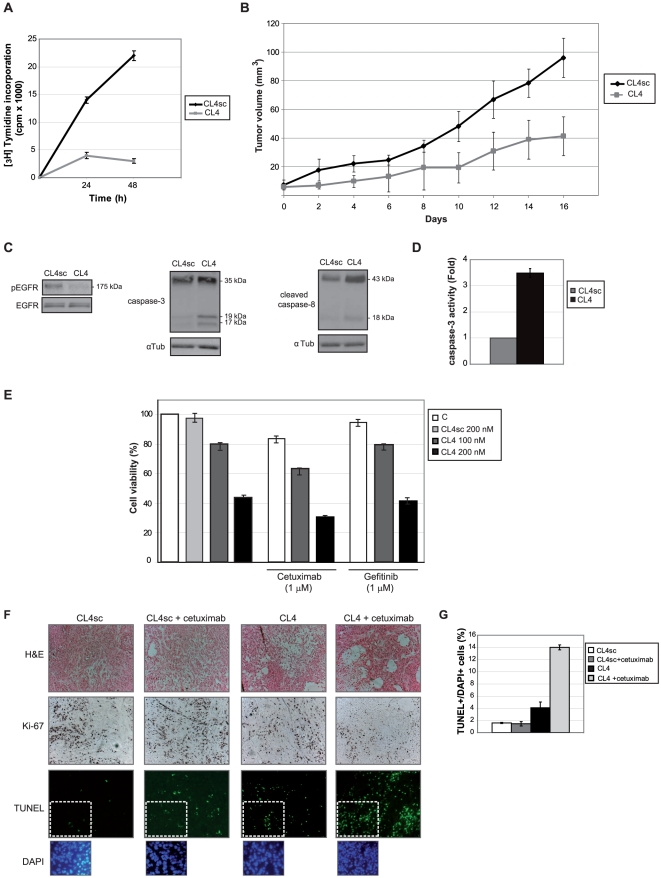
CL4 inhibits tumor growth. (A) A549 cells (2,000 cells/well in 24-well plates) were treated for 24 h or 48 h with CL4 or CL4sc (200 nM-final concentration) and proliferation was determined by [^3^H]-thymidine incorporation. Vertical bars indicate the standard deviation values. (B) Growth inhibition of tumors in a mouse xenograft model bearing EGFR-positive A549 cells upon CL4 treatment (57% at 16 days compared to CL4sc control group, *P*<0.01 by Mann-Whitney test). Day 0 marks the first day of injection. Data are shown as means ± s.e.m. (n = 8 tumors). (C) Three tumors per group selected randomly were excised, lysed, and the pooled lysates were immunoblotted with anti-pEGFR, anti-EGFR, anti-caspase-3, anti-cleaved caspase-8 and anti-αtubulin antibodies, as indicated. Molecular weights of indicated proteins are reported. (D) Cell lysates as in (C) were analyzed by caspase-3 fluorimetric assays. (E) A549 cells (4,000 cells/well in 96-well plates) were left untreated or treated for 24 h with CL4sc (200 nM-final concentration) or CL4 (100 and 200 nM-final concentration) alone or in combination with 1 µM cetuximab or 1 µM gefitinib. Cell viability was analyzed as reported in Methods and was expressed as percent of viable treated cells with respect to control untreated cells (indicated with “C”). Error bars depict means ± s.d. (n = 4). (F) Representative sections of tumors from the CL4sc, CL4sc plus cetuximab, CL4, and CL4 plus cetuximab groups (see “Materials and Methods” for details) stained with H&E, Ki-67 antibody and TUNEL, as indicated. DAPI counterstaining of the boxed regions is shown. Note reduction in cell density in the CL4-treated section stained with H&E. Magnification, 20× for H&E and Ki-67 and 40× for TUNEL and DAPI. (G) Percentage of TUNEL^+^/DAPI^+^ cells, values represent mean ± s.d. for 10 randomly selected fields.

We next assessed the efficiency of CL4 for its ability to inhibit cell proliferation *in vitro* and limit tumor growth *in vivo*. As shown, treating A549 cells with CL4 for 24 and 48 h completely blocks [^3^H]-thymidine incorporation (Fig. 6A). In addition, in A549-mouse xenografts a pronounced reduction in tumor volume was observed in the presence of CL4-treatment, leading at day 16 to 57% inhibition with respect to CL4sc control (Fig. 6B). According with the effects observed *in vitro*, CL4-treatment of xenograft tumors decreases the extent of EGFR tyrosine phosphorylation and activates caspase- 3 and -8 (Fig. 6C,D).

As a next step, we compared the inhibition effect on cell viability of the CL4 to that of two commercially available EGFR inhibitors that are currently in clinical use as anticancer therapeutics, gefitinib and cetuximab. In dose-dependent experiments (gefitinib, 0.1–10 µM and cetuximab, 0.05–1 µM), A549 cells resulted resistant at any concentration, even a high concentration of the above inhibitors ([25], [26], and our unpublished data). Interestingly, cells are highly sensitive to a 200 nM-final concentration CL4-treatment (Fig. 6E) and the same effect was observed on Calu1 and A431 (not shown). Further, as shown, the combined treatment of CL4 with cetuximab inhibited A549 cell viability more effectively than the treatment with each single agent alone, thus showing additive interactions. On the contrary, CL4 effectiveness was not improved when administered in combination with gefitinib. Most importantly, the synergy between CL4 and cetuximab was confirmed *in vivo* in mice xenografted with A549 cells (Fig. 6F,G). Indeed, the combination of CL4 and cetuximab decreased the number of proliferating Ki-67-positive cells and increased the number of apoptotic cells stained positively for terminal deoxynucleotidyl transferase mediated dUTP nick end labeling (TUNEL) more efficiently than the treatment of each inhibitor alone. Whether the aptamer and the antibody bind to different epitopes on the receptor, remain to be determined.

## Discussion

Here we developed and characterized a 2′-F Py RNA aptamer, named CL4, capable of binding and inhibiting EGFR.

We show that CL4 binds EGFR on tumor cell surface as well as the soluble extracellular domain of the receptor with a K_d_ of 10 nM, while it does not bind to the other members of the ErbB family, ErbB2, ErbB3 or ErbB4. It specifically binds to any cell types provided that EGFR is expressed on cell surface and inhibits both EGFR activation and EGFR-mediated signal pathways.

It has been clearly shown that EGFR monomers can pair and form heterodimers with other members of the ErbB family [4], [27], [28]. The presence of these heterodimers renders several EGFR inhibitors poorly efficient as therapeutics [29]. By binding either the EGFR monomer or the dimer CL4 may act by blocking the receptor activation *via* inhibition of either EGFR homodimers and heterodimers with cognate ErbB2 or ErbB3, thus irrespective of the ligand that causes receptors dimerization. Indeed, treatment of EGFR-positive cancer cells with CL4 strongly inhibits both the EGF-induced tyrosine phosphorylation of EGFR and ErbB2 and the Hrg-dependent tyrosine phosphorylation of EGFR and ErbB3. In all cases, inhibition is mediated by specific recognition of EGFR since CL4 has no effect on EGFR-negative cells.

The mitogen-activated protein kinase pathway is a major downstream signaling route of the EGFR/ErbB family and is an invariable target of all ErbB ligands [3], [4]. Consistently, in A549 and Calu1 cells, expressing both EGFR and ErbB3, CL4 strongly reduces phospho-ERK 1/2 induced by either EGF or Hrg stimulation. Conversely, a strong reduction of AKT activation following CL4-treatment was observed in the presence of Hrg stimulation of the cells but not of EGF stimulation *i.e.* when EGFR activation proceeds essentially through dimerization of EGFR with ErbB3. It is reported that PI3K couples directly with ErbB3 but indirectly with EGFR *via* Gab1 since PI3K docking sites are absent on EGFR and ErbB2, whereas, six sites are present on ErbB3 [2], [30]. This means that the EGFR-dependent activation of PI3K occurs mainly through dimerization of EGFR with ErbB3. Accordingly, a recent computational model of the ErbB signaling network identified ErbB3 as the key node in ligand-induced activation of the ErbB receptor-PI3K axis [24]. Thus, the CL4 ability to inhibit phospho-AKT only when induced by Hrg but not EGF, could be explained by an aptamer preferential inhibition of EGFR-EGFR with respect to EGFR-ErbB3 complexes induced by EGF stimulation.

The selective induction of cell death in cancer treatment is the goal of new therapeutic strategies. We found that, as a consequence of the EGFR inhibition, CL4 strongly inhibits the anti-apoptotic STAT3 and induces cell death selectively in EGFR-positive cancer cells by activation of caspase-3, caspase-8 and PARP. Notably, CL4 effectively induces apoptosis in a xenograft model of NSCLC and inhibits tumor growth up to 16-days treatment as assessed by tumor volumes. It is noteworthy that CL4 is protected from rapid nuclease degradation by the 2′-F Py modification thus increasing its half-life *in vivo* of several hours. Further it has been shown that this modification renders aptamers even less immunogenic than natural RNA [31].

Five EGFR inhibitors, two monoclonal antibodies (cetuximab, panitumumab) and three TKIs (erlotinib, gefitinib and lapatinib), have recently gained FDA approval in oncology. Despite clinical success, patients who initially respond to EGFR inhibitors may subsequently become refractory [10], [11], thus the attention has been directed towards alternative strategies, including the use of combined therapeutic protocols [10], [11]. Interestingly, CL4 strongly induces apoptosis of cancer cell lines that are resistant to those concentrations of gefitinib and cetuximab that reflect the mean steady-state plasma concentrations at the FDA-approved dosing level [32]. Further, the combined treatment of A549 cells with CL4 and cetuximab inhibits cell viability more effectively than the treatment with each single inhibitor, thus showing additive interactions.

Most importantly, CL4 and cetuximab show a clear synergy when used in combination in the treatment of A549 tumors *in vivo* as revealed by histological examination of the tumor sections for proliferating and apoptotic cells staining. Whether the epitopes recognized by the monoclonal antibody and the aptamer overlap remain to be ascertained.

Recently, RNA-aptamers have been selected against the purified ectodomain of EGFR [18], [19], [20]. No similarity of sequence exists between CL4 and these anti-EGFR aptamers. Further, despite they are high affinity ligands for EC-EGFR (K_d_ values in the range of 2–7 nM), no *in vivo* functional effect has been associated to the above aptamers.

In conclusion, the inhibitory properties of CL4 demonstrate its potential usefulness as a lead compound for the design of a new class of anticancer drugs and may become a valuable addition to the repertoire of inhibitors that target cancers that overexpress EGFR.

## Materials and Methods

### Ethics Statement

All the experimental procedures were approved by the Ethical Committee for the Animal Use (CESA) of the Istituto di Ricerche Genetiche Gaetano Salvatore (IRGS) and where communicated to the national authorities accordingly with national and European rules (permit number 1519).

### Cells and shRNA transfection

Growth conditions for human cell lines used (American Type Culture Collection) were previously reported: NSCLC A549, H460 and Calu1 [33], glioma U87MG and T98G [34], breast MCF7, SKBr3 and T47D [35]. NIH3T3 and NIH/EGFR are previously described [36].

For EGFR gene silencing, A549 (350,000 cells per 6 cm plate) were transfected with 6 µg of shRNAEGFR or shRNActrl (Open Biosystems) and Lipofectamine 2000 (Invitrogen).

### Immunoblot analysis

Immunoprecipitation and immunoblotting were performed as described [37]. The primary antibodies used were: anti-phospho-EGFR (Tyr1062), anti-ErbB3, anti-phospho-ErbB3 (Tyr1222), anti-ErbB2, anti-phospho-ERK1/2 (E10), anti-phospho-AKT (Ser473), anti-phospho-AKT (Thr308), anti-AKT, anti-phospho-STAT3 (Tyr705), anti-caspase-3, anti-caspase-8 (1C12) and anti-PARP, anti-cleaved Caspase-8 (18C8) (Cell Signaling); anti-ERK1 (C-16), anti-EGFR, anti-ErbB3 (C-17), anti-ErbB4 (C-18) (Santa Cruz Biotechnology); anti-phospho-tyrosine (4G10, Upstate Biotechnology Incorporated); anti-α tubulin (DM 1A) (Sigma). RTK antibody arrays (R&D Systems) were performed as recommended.

For ligand-dependent EGFR activation EGF and Hrg1β1 (indicated as Hrg), both from R&D Systems, were used.

### Cell viability assay and FACS analysis

Cell viability was assessed with CellTiter 96® AQueous One Solution cell Proliferation Assay (Promega) as recommended. To assess apoptosis, cells were stained with 2 µg/ml propidium iodide (PI, Sigma) for 30 min at 4°C and analyzed by FACS. For combined treatment with CL4, we used TRAIL (Alexis Biochemicals), paclytaxel, cisplatin (Sigma) and gefitinib (LC laboratories).

### Caspases 3, 8 and 9 activity measurement

50 µg-cell lysates were incubated at 37°C for 1 h with substrate of caspase-3 (Ac-DEVD-AFC), caspase-8 (Ac-IETD-AFC) or caspase-9 (Ac-LEHD-AFC) (Alexis Bichemicals) and the fluorescence from cleaved substrate was measured.

### Whole-cell SELEX and aptamers

A library of 2′F-Py RNAs containing a central stretch of 45 random nucleotides was subjected to a differential SELEX protocol against NSCLC. At each round, the positive selection step on A549 cells was preceded by one or two counterselection steps against H460 cells and the SELEX cycle was performed as described [34] .

CL4 and CL4sc, the scrambled sequence of CL4, were purchased from Sigma:

CL4: 5′ GCCUUAGUAACGUGCUUUGAUGUCGAUUCGACAGGAGGC 3′
CL4sc: 5′UUCGUACCGGGUAGGUUGGCUUGCACAUAGAACGUGUCA 3′

Before each treatment, the aptamers were subjected to a short denaturation-renaturation step (85°C for 5 min, snap-cooled on ice for 2 min, and allowed to warm up to 37°C). For cell treatment longer than 6 h, RNA concentrations were determined to ensure the continuous presence of at least 200 nM-concentration taking into account the 6 h-half life of the aptamer in 10% serum.

### Aptamer binding analysis

Binding of CL4 or CL4sc to cells was performed as described [34]. Filter binding analysis with the soluble extracellular domain of human EGFR and ErbB3 as targets (R&D Systems), was assessed by incubating 1 nM of radiolabeled aptamers with 1, 3.2, 10, 32, 100, 320 and 1000 nM of EC-EGFR or EC-ErbB3 for 15 min at 37°C in PBS supplemented with 0.01% bovine serum albumin. EC-EGFR and EC-ErbB3 are disulfide-linked homodimers; to assess the CL4 binding to the monomeric recombinant proteins, analysis was performed in the presence of 5 mM DTT.

### CL4-mediated affinity purification

CL4 or CL4sc were 3′-end biotinylated by terminal transferase and Biotin-ddUTP (Roche) and incubated in serum free culture medium at a 200 nM-final concentration on A549 cells for 30 min at RT. Cells were washed with PBS and lysed with 10 mM Tris-HCl pH 7.5 containing 200 mM NaCl, 5 mM EDTA, 0.1% Triton X-100, and protease inhibitors. 400 µg-cell extract was incubated with 200 µl-streptavidin beads (Pierce) in 0.4 ml lysis buffer at RT for 1 h with rotation. Following four washes with PBS, bound proteins were recovered with Laemmli buffer and analyzed by immunoblotting with anti-EGFR antibody.

### [^3^H]-Thymidine incorporation assay

A549 cells were treated for 24 h or 48 h with CL4 or CL4sc. During the final 6 h, cells were pulsed with 1 µCi/ml [^3^H]-thymidine (45 Ci/mmol) (Amersham-Pharmacia Biosciences) added in complete growth medium and incubated at 37°C. At the end of each pulse, cells were harvested and [^3^H]-thymidine incorporation was analyzed by a Beckman LS 1701 Liquid Scintillation Counter.

### *In vivo* experiments

Athymic CD-1 nude mice (nu/nu) were housed in a highly controlled microbiological environment, thus to guarantee specific pathogen free conditions. Mice were injected subcutaneously with 3×10^6^ (in 100 µl) *in vitro* propagated A549. Sixteen non-necrotic tumors of about 0.5 cm in diameter were randomly divided into two groups of eight mice as follows: group 1, CL4sc-treated; group 2, CL4-treated. Aptamers (200 pmols/injection) were injected intratumorally in 100-µl volumes three times a week for 16 days. During the study mice were daily monitored to avoid any sign of suffering. Tumors were measured every 2 days with calipers and tumor volume was calculated as follows: V_T_ = (WXLXH)×0.5236 (W, the shortest dimension; L, the longest dimension; H, the intermediate dimension). For combined treatment of CL4 and cetuximab, 24 non-necrotic tumors of about 0.5 cm in diameter were randomly divided into four groups of six mice as follows: group 1, CL4sc (200 pmols/intratumor injection three times a week for 21 days); group 2, CL4sc plus cetuximab (200 pmols/intratumor injection three times a week for 21 days, plus 25 µg cetuximab/intraperitoneal injection in 100-µl volumes once a week for the last 14 days); group 3, CL4 (200 pmols/intratumor injection three times a week for 21 days); group 4, CL4 plus cetuximab (200 pmols/intratumor injection three times a week for 21 days, plus 25 µg cetuximab/intraperitoneal injection in 100-µl volumes once a week for the last 14 days).

### Histology and immuno-histochemistry

Tumors were embedded in paraffin and sectioned at 6 µm. To inhibit the endogenous peroxidases, the sections were treated with 0.5% H_2_O_2_ in absolute methanol for 15 min at RT. For histological examinations, serial paraffin sections were stained with Harris hematoxylin and aqueous eosin (H&E, BDH Laboratory Supplies).

Cell proliferation was assessed by Ki-67 immunohistochemistry. The anti-human Ki-67 antibody (Epitomics) was 1∶500 diluted and immunostaining was done using the immunoperoxidase system of the “Vectastain ABC kit” (Vector) and the “DAB substrate kit for peroxidise” (Vector), according with the manufacturer's protocol.

### TUNEL assay

Apoptotic cell death in paraffin tumor tissue sections was detected using TUNEL staining. Sections were permeabilized with 0.1% Triton X-100, 0.1% sodium citrate solution and apoptosis was detected with in situ Cell Death fluorescein kit (Roche) according to manufacturer's procedure. All staining were finally counterstained with DAPI before mounting. Microscopy and imaging were performed in a Zeiss AxionPlan II epifluorescence (FluoArc) Microscope. The images were processed using Axion Vision software and edited by Image J software.

### Statistical analyses

We performed statistical analyses with GraphPad Prism.

## Supporting Information

Figure S1 **CL4 inhibits serum-dependent EGFR and ErbB3 phosphorylation.** A549 cells were serum starved for 18 h and then stimulated with culture medium supplemented with 20% FBS for 10 min in the presence of 200 nM CL4 or CL4sc and cell extracts were prepared. (A) 200 µg-lysates were incubated on RTK antibody arrays. Phosphorylation levels were determined by subsequent incubation with anti-phosphotyrosine horseradish peroxidase. The pixel intensity associated to the phosphorylation status of EGFR, ErbB2, ErbB3 and ErbB4, is reported. Quantitation was done as in Figure 2 and error bars depict means ± s.d. (n = 4). (B) Cell lysates were immunoblotted with anti-pEGFR, anti-pErbB3 and anti-αtubulin antibodies, as indicated. Values below the blots indicate signal levels relative to stimulated CL4sc control, arbitrarily set to 1 (labeled with asterisk). Quantitation was done as in Figure 2. Blots shown are representative of at least two independent experiments.
(EPS)

Figure S2 **CL4 binds different EGFR-positive cancer cell lines.** (A,C) Lysates from the indicated cell lines were immunoblotted with anti-EGFR, anti-ErbB2, anti-ErbB3 and anti-ErbB4 antibodies, as indicated. αtubulin was used as an internal control. Blots shown are representative of at least three independent experiments. (B,D) Binding of radiolabeled CL4 (100 and 200 nM) on the indicated cell lines. The results are expressed relative to the background binding detected with CL4sc used as a negative control. Error bars depict means ± s.d. (n = 3).
(EPS)

Figure S3 **CL4 inhibits cell viability in EGFR-positive cancer cell lines.** (A) Indicated cell lines (4,000 cells/well in 96-well plates) were left untreated or treated for 24 h with CL4 or CL4sc (200 nM-final concentration) and cell viability was analyzed as reported in Methods and expressed as percent of viable treated cells with respect to control untreated cells (indicated with “C”). Error bars depict means ± s.d. (n = 4). (B) A549 cells were left untreated or treated for 24 h with CL4 or CL4sc (200 nM-final concentration) alone or in combination with TRAIL, cisplatin and paclytaxel at the indicated concentrations. Cell viability was analyzed as in (A), error bars depict means ± s.d. (n = 4).
(EPS)
